# *N*′-[1-(5-Bromo-2-hydroxy­phen­yl)ethyl­idene]-3,4,5-trihydroxy­benzohydrazide dimethyl sulfoxide solvate trihydrate

**DOI:** 10.1107/S1600536810007002

**Published:** 2010-02-27

**Authors:** Nura Suleiman Gwaram, Hamid Khaledi, Hapipah Mohd Ali, Ward T. Robinson, Mahmood A. Abdulla

**Affiliations:** a Department of Chemistry, University of Malaya, 50603 Kuala Lumpur, Malaysia; b Department of Molecular Medicine, University of Malaya, 50603 Kuala Lumpur, Malaysia

## Abstract

The benzohydrazide mol­ecule in the title compound, C_15_H_13_BrN_2_O_5_·C_2_H_6_OS·3H_2_O, is almost planar with an r.m.s. deviation for the non-H atoms of 0.078 Å. The organic mol­ecules, water and dimethyl sulfoxide solvent mol­ecules are linked by N—H⋯O, O—H⋯O and O—H⋯S inter­molecular hydrogen bonds, forming zigzag chains along the *a* axis. Intra­molecular O—H⋯O and O—H⋯N hydrogen bonds also occur.

## Related literature

For the biological properties of 3,4,5-trihydroxy­benzoic acid (gallic acid) derivatives, see: Arunkumar *et al.* (2006 ▶); Saxena *et al.* (2008 ▶). For the crystal structures of Schiff bases derived from 3,4,5-trihydroxy­benzoyl­hydrazide, see: Abdul Alhadi *et al.* (2009 ▶); Khaledi *et al.* (2009 ▶).
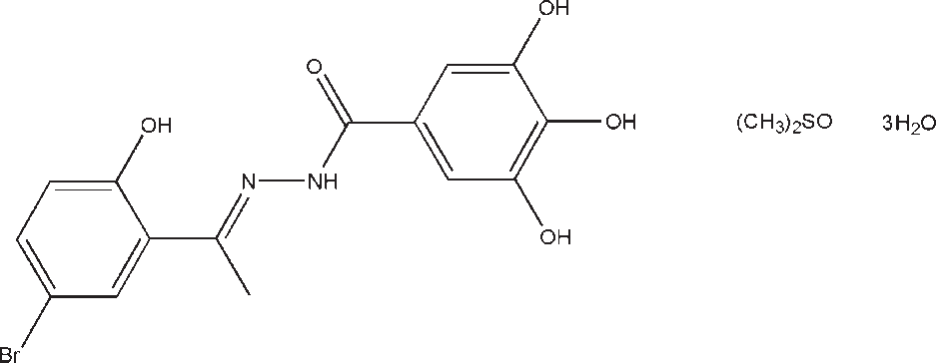


## Experimental

### 

#### Crystal data

C_15_H_13_BrN_2_O_5_·C_2_H_6_OS·3H_2_O
*M* *_r_* = 513.36
Monoclinic, 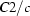

*a* = 21.5690 (15) Å
*b* = 7.0302 (4) Å
*c* = 28.4771 (18) Å
β = 103.061 (2)°
*V* = 4206.4 (5) Å^3^
*Z* = 8
Mo *K*α radiation
μ = 2.11 mm^−1^
*T* = 100 K
0.26 × 0.03 × 0.03 mm

#### Data collection

Bruker APEXII CCD area-detector diffractometer
Absorption correction: multi-scan (*SADABS*; Sheldrick, 1996 ▶) *T*_min_ = 0.610, *T*_max_ = 0.940
9543 measured reflections
3694 independent reflections
3052 reflections with *I* > 2σ(*I*)
*R*_int_ = 0.030

#### Refinement

*R*[*F* ^2^ > 2σ(*F* ^2^)] = 0.029
*wR*(*F* ^2^) = 0.066
*S* = 1.02
3694 reflections
307 parameters
11 restraints
H atoms treated by a mixture of independent and constrained refinement
Δρ_max_ = 0.34 e Å^−3^
Δρ_min_ = −0.30 e Å^−3^

### 

Data collection: *APEX2* (Bruker, 2007 ▶); cell refinement: *SAINT* (Bruker, 2007 ▶); data reduction: *SAINT*; program(s) used to solve structure: *SHELXS97* (Sheldrick, 2008 ▶); program(s) used to refine structure: *SHELXL97* (Sheldrick, 2008 ▶); molecular graphics: *X-SEED* (Barbour, 2001 ▶); software used to prepare material for publication: *publCIF* (Westrip, 2010 ▶).

## Supplementary Material

Crystal structure: contains datablocks I, global. DOI: 10.1107/S1600536810007002/hg2645sup1.cif

Structure factors: contains datablocks I. DOI: 10.1107/S1600536810007002/hg2645Isup2.hkl

Additional supplementary materials:  crystallographic information; 3D view; checkCIF report

## Figures and Tables

**Table 1:** Hydrogen-bond geometry (Å, °)

*D*—H⋯*A*	*D*—H	H⋯*A*	*D*⋯*A*	*D*—H⋯*A*
O1—H1⋯N1	0.82 (2)	1.80 (2)	2.531 (3)	149 (3)
O1—H1⋯O9	0.82 (2)	2.65 (2)	3.338 (2)	142 (3)
O2—H2*A*⋯O10^i^	0.84 (2)	1.88 (2)	2.703 (2)	168 (3)
O2—H2*A*⋯S^i^	0.84 (2)	2.86 (2)	3.5458 (19)	140 (2)
O2—H2*B*⋯O1^ii^	0.83 (2)	2.06 (2)	2.880 (2)	169 (3)
O3—H3*A*⋯O10	0.82 (2)	1.97 (2)	2.785 (3)	169 (3)
O3—H3*B*⋯O13^iii^	0.80 (2)	2.01 (2)	2.802 (2)	168 (3)
O4—H4*A*⋯O2^iv^	0.87 (2)	2.07 (2)	2.915 (3)	166 (3)
O4—H4*B*⋯O3	0.89 (2)	1.96 (2)	2.817 (3)	160 (3)
O12—H12⋯O3	0.84 (2)	1.91 (2)	2.751 (2)	179 (3)
O13—H13⋯O2^v^	0.80 (2)	1.88 (2)	2.651 (2)	160 (3)
O13—H13⋯O14	0.80 (2)	2.30 (3)	2.727 (2)	114 (2)
O14—H14⋯O9^vi^	0.82 (2)	1.87 (2)	2.682 (2)	169 (3)
N2—H2*N*⋯O4	0.87 (1)	2.24 (1)	3.113 (3)	179 (3)

Symmetry codes: (i) 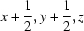; (ii) 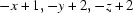; (iii) 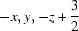; (iv) 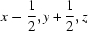; (v) 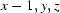; (vi) 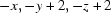.

**Fig. 1.**
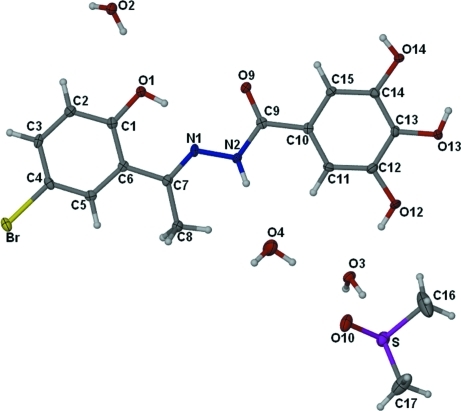
Thermal ellipsoid plot (Barbour, 2001) of the title compound at 50% probability level. Hydrogen atoms are drawn as spheres of arbitrary radius.

**Fig. 2.**
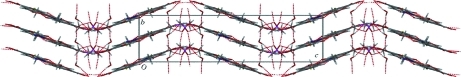
Zigzag chain along α axis formed by intermolecular hydrogen bonding

## supplementary crystallographic information

### Experimental

A mixture of 5-bromo-2-hydroxyacetophenone (2.15 g, 10 mmol) and 3,4,5-trihydroxybenzoylhydrazine(1.84 g, 10 mmol) was heated in ethanol (70 ml) for 3 h. The solution was then cooled and the solid product formed weas filtered off, washed with cold ethanol,and dried over silica gel. Crystals of the title compound were obtained by slow evaporation of a DMSO solution at room temperature.

### Refinement

C-bound hydrogen atoms were placed at calculated positions (C–H 0.95 Å), and were treated as riding on their parent carbon atoms, with U(H) set to 1.2Ueq(C). The nitrogen- and oxygen-bound H atoms were located in a difference Fourier map, and were refined with distance restraints of N–H 0.88±0.01 and O–H 0.84±0.02 Å.

### Crystal data

**Table:**

C_15_H_13_BrN_2_O_5_·C_2_H_6_OS·3H_2_O	*F*(000) = 2112
*M**_r_* = 513.36	*D*_x_ = 1.621 Mg m^−^^3^
Monoclinic, *C*2/*c*	Mo *K*α radiation, λ = 0.71073 Å
Hall symbol: -C 2yc	Cell parameters from 3053 reflections
*a* = 21.5690 (15) Å	θ = 2.7–27.9°
*b* = 7.0302 (4) Å	µ = 2.11 mm^−^^1^
*c* = 28.4771 (18) Å	*T* = 100 K
β = 103.061 (2)°	Needle, colourless
*V* = 4206.4 (5) Å^3^	0.26 × 0.03 × 0.03 mm
*Z* = 8	

### Data collection

**Table:**

Bruker APEXII CCD area-detector diffractometer	3694 independent reflections
Radiation source: fine-focus sealed tube	3052 reflections with *I* > 2σ(*I*)
graphite	*R*_int_ = 0.030
phi and ω scans	θ_max_ = 25.0°, θ_min_ = 2.2°
Absorption correction: multi-scan (*SADABS*; Sheldrick, 1996)	*h* = −25→25
*T*_min_ = 0.610, *T*_max_ = 0.940	*k* = −8→5
9543 measured reflections	*l* = −33→33

### Refinement

**Table:**

Refinement on *F*^2^	Primary atom site location: structure-invariant direct methods
Least-squares matrix: full	Secondary atom site location: difference Fourier map
*R*[*F*^2^ > 2σ(*F*^2^)] = 0.029	Hydrogen site location: inferred from neighbouring sites
*wR*(*F*^2^) = 0.066	H atoms treated by a mixture of independent and constrained refinement
*S* = 1.02	*w* = 1/[σ^2^(*F*_o_^2^) + (0.0311*P*)^2^ + 3.5353*P*] where *P* = (*F*_o_^2^ + 2*F*_c_^2^)/3
3694 reflections	(Δ/σ)_max_ = 0.001
307 parameters	Δρ_max_ = 0.34 e Å^−^^3^
11 restraints	Δρ_min_ = −0.30 e Å^−^^3^

### Special details

**Table:**

Geometry. All esds (except the esd in the dihedral angle between two l.s. planes) are estimated using the full covariance matrix. The cell esds are taken into account individually in the estimation of esds in distances, angles and torsion angles; correlations between esds in cell parameters are only used when they are defined by crystal symmetry. An approximate (isotropic) treatment of cell esds is used for estimating esds involving l.s. planes.
Refinement. Refinement of *F*^2^ against ALL reflections. The weighted *R*-factor wR and goodness of fit *S* are based on *F*^2^, conventional *R*-factors *R* are based on *F*, with *F* set to zero for negative *F*^2^. The threshold expression of *F*^2^ > σ(*F*^2^) is used only for calculating *R*-factors(gt) etc. and is not relevant to the choice of reflections for refinement. *R*-factors based on *F*^2^ are statistically about twice as large as those based on *F*, and *R*- factors based on ALL data will be even larger.

### Fractional atomic coordinates and isotropic or equivalent isotropic displacement parameters (Å^2^)

**Table:**

	*x*	*y*	*z*	*U*_iso_*/*U*_eq_	
Br	0.525034 (11)	1.15033 (4)	1.066977 (9)	0.01977 (9)	
S	0.15785 (3)	0.18073 (10)	0.73419 (2)	0.02470 (17)	
O1	0.25101 (8)	1.0736 (3)	1.07882 (6)	0.0201 (4)	
H1	0.2293 (12)	1.052 (4)	1.0519 (7)	0.030*	
O2	0.78049 (8)	0.7413 (3)	0.83940 (6)	0.0205 (4)	
H2A	0.7527 (12)	0.791 (4)	0.8174 (8)	0.031*	
H2B	0.7763 (14)	0.801 (4)	0.8636 (8)	0.031*	
O3	0.12345 (8)	0.6895 (3)	0.77600 (6)	0.0193 (4)	
H3A	0.1436 (13)	0.590 (3)	0.7769 (11)	0.029*	
H3B	0.1157 (13)	0.721 (4)	0.7481 (7)	0.029*	
O4	0.19398 (10)	0.9307 (3)	0.84748 (7)	0.0382 (5)	
H4A	0.2210 (14)	1.012 (4)	0.8408 (12)	0.057*	
H4B	0.1779 (16)	0.866 (4)	0.8207 (9)	0.057*	
O9	0.11275 (8)	0.9528 (3)	1.00576 (6)	0.0188 (4)	
O10	0.18508 (9)	0.3495 (3)	0.76525 (6)	0.0288 (5)	
O12	0.00733 (8)	0.7056 (3)	0.80056 (6)	0.0302 (5)	
H12	0.0426 (10)	0.702 (5)	0.7930 (11)	0.045*	
O13	−0.10287 (8)	0.7412 (3)	0.82391 (6)	0.0210 (4)	
H13	−0.1337 (11)	0.749 (4)	0.8352 (10)	0.032*	
O14	−0.11122 (8)	0.8463 (3)	0.91444 (6)	0.0213 (4)	
H14	−0.1073 (14)	0.900 (4)	0.9405 (7)	0.032*	
N1	0.22497 (9)	0.9902 (3)	0.99005 (7)	0.0148 (5)	
N2	0.17589 (9)	0.9350 (3)	0.95289 (7)	0.0146 (5)	
H2N	0.1813 (11)	0.933 (4)	0.9235 (5)	0.018*	
C1	0.31207 (11)	1.0888 (4)	1.07379 (8)	0.0156 (5)	
C2	0.35764 (12)	1.1406 (4)	1.11456 (9)	0.0186 (6)	
H2	0.3452	1.1618	1.1441	0.022*	
C3	0.42073 (11)	1.1617 (4)	1.11280 (9)	0.0175 (6)	
H3	0.4516	1.1992	1.1407	0.021*	
C4	0.43828 (11)	1.1272 (3)	1.06974 (9)	0.0156 (5)	
C5	0.39418 (11)	1.0743 (3)	1.02900 (9)	0.0150 (5)	
H5	0.4074	1.0523	0.9998	0.018*	
C6	0.32987 (11)	1.0524 (3)	1.02996 (8)	0.0135 (5)	
C7	0.28342 (11)	0.9907 (3)	0.98599 (8)	0.0143 (5)	
C8	0.30638 (12)	0.9305 (4)	0.94239 (9)	0.0199 (6)	
H8A	0.2713	0.8716	0.9190	0.030*	
H8B	0.3411	0.8386	0.9519	0.030*	
H8C	0.3218	1.0420	0.9278	0.030*	
C9	0.11782 (11)	0.9227 (3)	0.96425 (9)	0.0148 (5)	
C10	0.06185 (11)	0.8702 (3)	0.92563 (8)	0.0131 (5)	
C11	0.06462 (11)	0.8147 (4)	0.87924 (8)	0.0157 (6)	
H11	0.1045	0.8087	0.8703	0.019*	
C12	0.00928 (12)	0.7683 (4)	0.84614 (8)	0.0172 (6)	
C13	−0.04955 (11)	0.7827 (4)	0.85844 (8)	0.0146 (5)	
C14	−0.05204 (11)	0.8373 (4)	0.90472 (8)	0.0142 (5)	
C15	0.00347 (11)	0.8764 (3)	0.93839 (9)	0.0145 (5)	
H15	0.0017	0.9078	0.9705	0.017*	
C16	0.07421 (15)	0.1949 (6)	0.72909 (17)	0.0685 (13)	
H16A	0.0645	0.1626	0.7601	0.103*	
H16B	0.0525	0.1053	0.7044	0.103*	
H16C	0.0596	0.3244	0.7199	0.103*	
C17	0.15995 (19)	0.2421 (5)	0.67432 (11)	0.0490 (10)	
H17A	0.1387	0.3648	0.6661	0.074*	
H17B	0.1380	0.1441	0.6522	0.074*	
H17C	0.2043	0.2514	0.6715	0.074*	

### Atomic displacement parameters (Å^2^)

**Table:**

	*U*^11^	*U*^22^	*U*^33^	*U*^12^	*U*^13^	*U*^23^
Br	0.01221 (13)	0.01949 (15)	0.02680 (15)	−0.00176 (11)	0.00269 (10)	−0.00108 (12)
S	0.0249 (4)	0.0245 (4)	0.0222 (4)	0.0041 (3)	0.0002 (3)	−0.0006 (3)
O1	0.0126 (9)	0.0320 (11)	0.0155 (9)	−0.0023 (8)	0.0028 (7)	−0.0048 (9)
O2	0.0159 (10)	0.0290 (12)	0.0152 (9)	0.0006 (8)	0.0008 (8)	−0.0027 (8)
O3	0.0213 (10)	0.0236 (11)	0.0122 (9)	0.0033 (8)	0.0024 (8)	0.0000 (8)
O4	0.0409 (13)	0.0487 (15)	0.0254 (11)	−0.0210 (11)	0.0085 (10)	−0.0066 (11)
O9	0.0167 (9)	0.0261 (11)	0.0130 (9)	−0.0026 (8)	0.0022 (7)	−0.0036 (8)
O10	0.0270 (10)	0.0291 (11)	0.0246 (10)	0.0053 (9)	−0.0059 (8)	−0.0063 (9)
O12	0.0153 (9)	0.0640 (15)	0.0116 (9)	−0.0023 (10)	0.0038 (8)	−0.0127 (9)
O13	0.0102 (9)	0.0382 (12)	0.0145 (9)	−0.0028 (9)	0.0025 (7)	−0.0065 (9)
O14	0.0132 (8)	0.0364 (12)	0.0155 (9)	−0.0028 (8)	0.0058 (7)	−0.0116 (9)
N1	0.0130 (11)	0.0138 (11)	0.0153 (11)	−0.0025 (9)	−0.0017 (8)	−0.0010 (9)
N2	0.0137 (10)	0.0193 (12)	0.0102 (10)	−0.0011 (9)	0.0011 (8)	−0.0016 (10)
C1	0.0152 (13)	0.0142 (13)	0.0168 (13)	−0.0005 (10)	0.0021 (10)	0.0012 (11)
C2	0.0199 (13)	0.0199 (14)	0.0160 (13)	−0.0009 (12)	0.0040 (10)	−0.0032 (12)
C3	0.0174 (13)	0.0161 (14)	0.0155 (12)	−0.0001 (11)	−0.0036 (10)	−0.0021 (11)
C4	0.0116 (12)	0.0113 (13)	0.0236 (14)	−0.0015 (10)	0.0033 (10)	0.0012 (11)
C5	0.0182 (13)	0.0113 (13)	0.0161 (13)	−0.0006 (11)	0.0053 (10)	0.0016 (11)
C6	0.0161 (12)	0.0098 (13)	0.0138 (12)	0.0018 (11)	0.0020 (10)	0.0010 (10)
C7	0.0168 (13)	0.0124 (13)	0.0126 (12)	0.0007 (10)	0.0009 (10)	0.0013 (10)
C8	0.0169 (13)	0.0268 (15)	0.0157 (13)	−0.0021 (12)	0.0028 (11)	−0.0017 (12)
C9	0.0162 (13)	0.0096 (13)	0.0182 (13)	0.0012 (11)	0.0031 (10)	0.0018 (11)
C10	0.0148 (12)	0.0088 (13)	0.0152 (12)	0.0000 (10)	0.0021 (10)	0.0013 (11)
C11	0.0129 (12)	0.0210 (15)	0.0143 (12)	0.0021 (11)	0.0053 (10)	0.0001 (11)
C12	0.0195 (13)	0.0204 (14)	0.0116 (12)	0.0007 (11)	0.0034 (10)	−0.0012 (11)
C13	0.0123 (12)	0.0174 (14)	0.0127 (12)	−0.0004 (10)	−0.0003 (10)	0.0004 (11)
C14	0.0137 (12)	0.0131 (13)	0.0166 (12)	−0.0017 (11)	0.0051 (10)	−0.0011 (11)
C15	0.0163 (12)	0.0149 (14)	0.0127 (12)	−0.0004 (11)	0.0039 (10)	−0.0033 (11)
C16	0.0242 (18)	0.065 (3)	0.112 (4)	−0.0043 (18)	0.007 (2)	−0.042 (3)
C17	0.084 (3)	0.034 (2)	0.0243 (16)	−0.009 (2)	0.0029 (17)	−0.0009 (15)

### Geometric parameters (Å, °)

**Table:**

Br—C4	1.898 (2)	C2—H2	0.9500
S—O10	1.516 (2)	C3—C4	1.384 (3)
S—C17	1.769 (3)	C3—H3	0.9500
S—C16	1.780 (3)	C4—C5	1.375 (3)
O1—C1	1.361 (3)	C5—C6	1.402 (3)
O1—H1	0.818 (17)	C5—H5	0.9500
O2—H2A	0.839 (17)	C6—C7	1.481 (3)
O2—H2B	0.830 (17)	C7—C8	1.498 (3)
O3—H3A	0.823 (17)	C8—H8A	0.9800
O3—H3B	0.804 (17)	C8—H8B	0.9800
O4—H4A	0.867 (18)	C8—H8C	0.9800
O4—H4B	0.889 (18)	C9—C10	1.484 (3)
O9—C9	1.230 (3)	C10—C15	1.388 (3)
O12—C12	1.363 (3)	C10—C11	1.391 (3)
O12—H12	0.837 (18)	C11—C12	1.383 (3)
O13—C13	1.365 (3)	C11—H11	0.9500
O13—H13	0.803 (17)	C12—C13	1.394 (3)
O14—C14	1.368 (3)	C13—C14	1.385 (3)
O14—H14	0.821 (17)	C14—C15	1.382 (3)
N1—C7	1.292 (3)	C15—H15	0.9500
N1—N2	1.373 (3)	C16—H16A	0.9800
N2—C9	1.365 (3)	C16—H16B	0.9800
N2—H2N	0.870 (10)	C16—H16C	0.9800
C1—C2	1.389 (3)	C17—H17A	0.9800
C1—C6	1.410 (3)	C17—H17B	0.9800
C2—C3	1.381 (3)	C17—H17C	0.9800
			
O10—S—C17	106.27 (14)	H8A—C8—H8B	109.5
O10—S—C16	104.83 (14)	C7—C8—H8C	109.5
C17—S—C16	98.9 (2)	H8A—C8—H8C	109.5
C1—O1—H1	106 (2)	H8B—C8—H8C	109.5
H2A—O2—H2B	103 (3)	O9—C9—N2	120.2 (2)
H3A—O3—H3B	105 (3)	O9—C9—C10	121.4 (2)
H4A—O4—H4B	107 (3)	N2—C9—C10	118.4 (2)
C12—O12—H12	115 (2)	C15—C10—C11	119.6 (2)
C13—O13—H13	110 (2)	C15—C10—C9	115.6 (2)
C14—O14—H14	107 (2)	C11—C10—C9	124.8 (2)
C7—N1—N2	122.0 (2)	C12—C11—C10	119.8 (2)
C9—N2—N1	115.11 (19)	C12—C11—H11	120.1
C9—N2—H2N	123.8 (17)	C10—C11—H11	120.1
N1—N2—H2N	119.9 (17)	O12—C12—C11	124.2 (2)
O1—C1—C2	116.8 (2)	O12—C12—C13	115.4 (2)
O1—C1—C6	122.9 (2)	C11—C12—C13	120.4 (2)
C2—C1—C6	120.3 (2)	O13—C13—C14	122.5 (2)
C3—C2—C1	121.0 (2)	O13—C13—C12	118.0 (2)
C3—C2—H2	119.5	C14—C13—C12	119.5 (2)
C1—C2—H2	119.5	O14—C14—C15	123.5 (2)
C2—C3—C4	118.8 (2)	O14—C14—C13	116.4 (2)
C2—C3—H3	120.6	C15—C14—C13	120.1 (2)
C4—C3—H3	120.6	C14—C15—C10	120.5 (2)
C5—C4—C3	121.3 (2)	C14—C15—H15	119.8
C5—C4—Br	119.25 (18)	C10—C15—H15	119.8
C3—C4—Br	119.44 (18)	S—C16—H16A	109.5
C4—C5—C6	120.8 (2)	S—C16—H16B	109.5
C4—C5—H5	119.6	H16A—C16—H16B	109.5
C6—C5—H5	119.6	S—C16—H16C	109.5
C5—C6—C1	117.8 (2)	H16A—C16—H16C	109.5
C5—C6—C7	119.9 (2)	H16B—C16—H16C	109.5
C1—C6—C7	122.3 (2)	S—C17—H17A	109.5
N1—C7—C6	114.4 (2)	S—C17—H17B	109.5
N1—C7—C8	125.8 (2)	H17A—C17—H17B	109.5
C6—C7—C8	119.8 (2)	S—C17—H17C	109.5
C7—C8—H8A	109.5	H17A—C17—H17C	109.5
C7—C8—H8B	109.5	H17B—C17—H17C	109.5

### Hydrogen-bond geometry (Å, °)

**Table:**

*D*—H···*A*	*D*—H	H···*A*	*D*···*A*	*D*—H···*A*
O1—H1···N1	0.82 (2)	1.80 (2)	2.531 (3)	149 (3)
O1—H1···O9	0.82 (2)	2.65 (2)	3.338 (2)	142 (3)
O2—H2A···O10^i^	0.84 (2)	1.88 (2)	2.703 (2)	168 (3)
O2—H2A···S^i^	0.84 (2)	2.86 (2)	3.5458 (19)	140 (2)
O2—H2B···O1^ii^	0.83 (2)	2.06 (2)	2.880 (2)	169 (3)
O3—H3A···O10	0.82 (2)	1.97 (2)	2.785 (3)	169 (3)
O3—H3B···O13^iii^	0.80 (2)	2.01 (2)	2.802 (2)	168 (3)
O4—H4A···O2^iv^	0.87 (2)	2.07 (2)	2.915 (3)	166 (3)
O4—H4B···O3	0.89 (2)	1.96 (2)	2.817 (3)	160 (3)
O12—H12···O3	0.84 (2)	1.91 (2)	2.751 (2)	179 (3)
O13—H13···O2^v^	0.80 (2)	1.88 (2)	2.651 (2)	160 (3)
O13—H13···O14	0.80 (2)	2.30 (3)	2.727 (2)	114 (2)
O14—H14···O9^vi^	0.82 (2)	1.87 (2)	2.682 (2)	169 (3)
N2—H2N···O4	0.87 (1)	2.24 (1)	3.113 (3)	179 (3)

Symmetry codes: (i) *x*+1/2, *y*+1/2, *z*; (ii) −*x*+1, −*y*+2, −*z*+2; (iii) −*x*, *y*, −*z*+3/2; (iv) *x*−1/2, *y*+1/2, *z*; (v) *x*−1, *y*, *z*; (vi) −*x*, −*y*+2, −*z*+2.
